# Characterization of Swedish *Campylobacter coli* clade 2 and clade 3 water isolates

**DOI:** 10.1002/mbo3.583

**Published:** 2018-02-09

**Authors:** Anna Nilsson, Astrid Skarp, Cecilia Johansson, René Kaden, Lars Engstrand, Hilpi Rautelin

**Affiliations:** ^1^ Department of Medical Sciences Clinical Microbiology Uppsala University Uppsala Sweden; ^2^ Department of Microbiology, Tumor and Cell Biology Karolinska Institute and Science for Life Laboratory Clinical Genomics Stockholm Sweden; ^3^ Present address: School of Engineering and Applied Science, Biology and Medical Laboratory Research Rotterdam University of Applied Sciences Rotterdam The Netherlands

**Keywords:** *Campylobacter coli*, phenotypic identification, waterborne pathogens, whole‐genome sequencing

## Abstract

*Campylobacter jejuni* and *Campylobacter coli* are important bacterial enteropathogens. Poultry is the best‐known reservoir for *Campylobacter* infection but natural bodies of water have also been shown to be important pathways for transmission. *Campylobacter* can survive in cold water but most of the studies have focused on *C*. *jejuni* only. In this paper, we take a closer look at the biology and water survival strategies of *C*. *coli*. Eight *C*. *coli* isolates cultivated from raw (incoming) surface water at water plants in Sweden were characterized using whole‐genome sequencing and phenotypical assays. Phylogenetic analysis assigned the Swedish water isolates to clades 2 and 3, known to include *C*. *coli* of environmental origin. In addition, 53 earlier published sequences of *C*. *coli* clade 2 and 3 from environmental waters were included for in silico analyses. Generally, clade 2 isolates had larger genomes, which included a functional tricarballylate utilization locus, while clade 3 isolates contained different genes involved in oxidative stress as well as putative virulence factors. The Swedish water isolates of clade 2 formed large, blurry bacterial colonies on agar, whereas clade 3 colonies were smaller. All Swedish isolates were motile, but clade 3 isolates formed larger motility zones on soft agar, and none of these isolates produced biofilm. Although water survival varied between the analyzed isolates, there were hardly any clade‐specific significant differences. Our results highlight the diversity of *C*. *coli* in general, and show differences in metabolic capabilities and ways to handle oxidative stress between clade 2 and 3 water isolates.

## 1 INTRODUCTION

*Campylobacter* is the most common cause of bacterial gastroenteritis in many Western countries and *Campylobacter jejuni* and *Campylobacter coli* cause the majority of the infections (Curtis, Hill, Wilcock, & Charlebois, 2014). Major reservoirs for these two *Campylobacter* species are warm‐blooded animals such as poultry, ruminants, and pigs and transmission to humans is thought mainly to occur via handling and eating poultry and drinking unpasteurized milk (Skirrow, 1991; Thomas, Gibson, Hill, & Mabey, 1998). Other pathways for transmission include environmental reservoirs, such as untreated drinking water from private water supplies and recreational surface water (Hänninen et al., 2003; Kapperud, 2003; Schönberg‐Norio et al., 2004). The transmission of campylobacters via environmental pathways is likely reflected by the survivability rather than multiplication of the organism (Bolton, 2015). Studies on different *C. jejuni* isolates have shown that the survival time in water at a low temperature varies between 2 weeks and 4 months (Cools et al., 2003; Rollins & Colwell, 1986; Trigui, Thibodeau, Fravalo, Letellier, & Faucher, 2015). It has also been noted that *C. jejuni* isolates derived from various sources exhibit different water survival potential (Bronowski et al., 2017; Buswell et al., 1998; Cools et al., 2003; Jones, Sutcliffe, & Curry, 1991). These differences have been suggested to be a consequence of variation in the genetic content between the isolates (Trigui et al., 2015). However, the majority of the *Campylobacter* water survival studies have mainly focused on *C. jejuni* and only a few have compared *C. jejuni* and *C*. *coli* (Korhonen & Martikainen, 1991; Thomas, Hill, & Mabey, 1999).

Molecular typing and whole‐genome sequencing have shown the population structure of *C*. *coli* to be divided into three clonally related clades associated with ecologically distinct niches, unlike *C. jejuni,* which consists of many clonal complexes (Sheppard, McCarthy, Falush, & Maiden, 2008; Sheppard et al., 2013). Only two MLST‐based clonal complexes have been found in *C*. *coli* and they both belong to *C*. *coli* clade 1 (Sheppard & Maiden, 2015). *C*. *coli* clades 2 and 3 seem to include the majority of isolates from environmental origins and show no organized clonal complex structure as opposed to *C*. *coli* clade 1 and many *C. jejuni* (Sheppard et al., 2013). The majority of clinical and farm animal *C*. *coli* isolates belong to the two clade 1 clonal complexes and clade 1 is associated with agriculture‐adapted *C*. *coli* with up to 23% of the genome originating from *C. jejuni* (Sheppard et al., 2013). Furthermore, whole‐genome sequencing can be applied to study the underlying genetic interstrain differences responsible for phenotypical traits as shown for *C. jejuni* (Lehri et al., 2015; Revez et al., 2011). Thus, combining genomics data with phenotypic observations may lead to a better understanding of the biology of these organisms.

For survival of *Campylobacter* in the environment, such as water, strategies for handling different types of stress are needed. For this purpose, motility and biofilm production may be used (Costerton et al., 1987; Ottemann & Miller, 1997). Motility has been shown to be crucial for host colonization (Black, Levine, Clements, Hughes, & Blaser, 1988; Guerry, 2007; Malik‐Kale et al., 2007) allowing the bacteria to move through the mucus layer to reach the epithelium (Szymanski, King, Haardt, & Armstrong, 1995). Moreover, motility has been shown to be an important prerequisite in the biofilm formation of *C. jejuni* (Kalmokoff et al., 2006). Biofilms can provide a protective environment and allow for dispersal of the bacteria, improving their chances to survive under harsh conditions. Biofilm studies focused on *C. jejuni* have shown differences between isolates belonging to different lineages (Asakura et al., 2012; Pascoe et al., 2015; Revez et al., 2011) and Sulaeman et al. (2010) showed variation in the ability of *C*. *coli* to initiate biofilm formation, which was generally lower than for *C. jejuni* (Sulaeman et al., 2010).

In this study, we characterized *C*. *coli* isolates cultivated from raw surface water at water plants in order to better understand the biology of these organisms and their survival strategies in water. We used whole‐genome sequencing and various phenotypical assays to determine clade assignment of the isolates and to reveal phenotypic characteristics typical for the isolates of the different clades. We show differences in metabolic capabilities and ways to handle oxidative stress between the two clades.

## 2 MATERIALS AND METHODS

### 2.1 Bacterial isolates

The eight bacterial isolates characterized in this study were collected in 2000 by the National Food Agency from raw (incoming) water samples taken from surface water at water plants in Sweden (Table 1). Originally, 200 ml of water was filtered through a 0.22 μm filter and the filter was thereafter incubated in preheated enrichment broth (Preston broth) at 37°C for 24 hr in a microaerobic environment. The broth was then cultured on CCDA plates (Blood‐free campylobacter‐selective media) to isolate *Campylobacter*. The isolates were identified as *C*. *coli* using Maldi‐ToF Biotyping (Microflex, Bruker, Billerica, Massachusetts, US). In addition, the earlier characterized clinical *C*. *coli* 76339 (Skarp‐de Haan et al., 2014) and *C. jejuni* 76577 (Revez et al., 2011) isolates, and the *C. jejuni* reference strain NCTC 11168 and the *C*. *coli* clade 1 reference strain LMG 6440 were used as experimental controls. All experiments were performed on isolates cultured directly from −80°C freezer to keep the passage number low.

**Table 1:** Water sample collection information and genetic description for the *Campylobacter coli* water isolates

*C*. *coli* isolate	Information on water sample collection			Genetic description				
	Location	Date (Month, year)	Water temp. (°C)	Clade	No. of contigs	Genome size^a^ (Mbp)	Plasmids	Accession no.
VA6	Norrköping	March, 2000	4	2	71	1.76	Yes^b^	MPIQ00000000
VA7	Norrköping	April, 2000	4.3	3	26	1.66	No	MPIR00000000
VA8	Eskilstuna	March, 2000	2	2	48	1.83	No	MPIS00000000
VA15	Eskilstuna	April, 2000	11.2	3	24	1.68	No	MPIT00000000
VA24	Botkyrka	June, 2000	12.9	2	44	1.83	Yes^b^	MPIU00000000
VA37	Trollhättan	October, 2000	10.7	2	51	1.86	Yes^b^	MPIV00000000
VA38	Norrköping	October, 2000	12.5	3	31	1.77	No	MPIW00000000
VA46	Trollhättan	November, 2000	7.5	2	52	1.95	Yes^b^	MPIX00000000

a Plasmids included in genome size.

b Number and size of plasmids were not exactly determined, but were below 20,000 bp.

### 2.2 Genomics

The isolates were cultured on blood agar (Columbia based agar plates supplemented with 5% horse blood; Oxoid, Basingstoke, UK) and incubated at 42°C for 24–48 hr in a microaerobic atmosphere (CampyGen, Oxoid). The DNA was extracted from pure bacterial cultures with MagNa Pure Compact Nucleic Acid isolation Kit I (Roche, Penzberg, Germany) according to the manufacturer's protocol version 12. An Illumina HiSeq 2500 platform with a 2 × 300 paired end run was applied for whole‐genome sequencing. The reads were assembled to contigs in Geneious (version 8.1.5.) (Kearse et al., 2012) with the Mira plugin (version 1.0.1.) and merging contigs were assembled with Geneious. Gegenees (Ågren, Sundström, Håfström, & Segerman, 2012) was used with a threshold of 20% to align the whole‐genome sequences of the Swedish *C*. *coli* water isolates with publicly available sequences of *C*. *coli* (Table S1) to reconstruct the phylogeny. For individual genes *tcuCAB, motAB,* and *pseA,* alignment and phylogenetic analysis were done in CLC Main Workbench (Qiagen, Hilden, Germany) using standard program settings. Additional searches in the whole‐genome sequences were performed in Bionumerics (version 7.6.1 created by Applied Maths NV. Available from http://www.applied-maths.com) using the BLAST function. The assembled sequences were annotated by RAST (Aziz et al., 2008) and the translated coding sequences (tCDS) were extracted. A reciprocal BLASTp query was performed using an e‐value of 1e‐5 and the OrthAgogue (Ekseth, Kuiper, & Mironov, 2014) and MCL‐edge tools (Enright, Van Dongen, & Ouzounis, 2002) were used to determine orthologous clusters. The predicted protein sequence of the tricarballylate gene locus derived from the isolate VA8 was blasted using Geneious (Kearse et al., 2012).

Plasmids were isolated from the *C*. *coli* water isolates using the GeneJet Plasmid Miniprep Kit (Thermo Fisher Scientific, Waltham, Massachusetts, US) according to the manufacturer's protocol. The plasmids were visualized with gel electrophoresis using a 0.7% agarose gel.

### 2.3 Phenotyping

#### 2.3.1 Preparation of bacterial suspensions

Bacteria were cultured for 17–18 hr in Brucella broth (Becton, Dickinson and Company, Franklin Lakes, New Jersey, US) at 42°C in a microaerobic atmosphere. The bacteria were collected by centrifuging at 8,000*g* for 5 min, removing the supernatant and resuspending the pellets in phosphate‐buffered saline (PBS) or nonsupplemented medium RPMI 1640 without phenol red or l‐glutamine (RPMI 1640; SVA, Uppsala, Sweden) to the desired concentration. All phenotypic experiments described below were performed in duplicate three times unless otherwise stated.

#### 2.3.2 Water survival

Bacteria suspended in PBS were added to 5 ml autoclaved tap water (pH 7) to a final concentration of 10^7^ cfu/ml and incubated at 4°C under aerobic conditions in the dark in a standard incubator. Samples for viable counts were taken at the start of the experiments and on days 2, 4, 6, and 8 using a 1:10 dilution series, plated out on blood agar and incubated at 42°C in a microaerobic atmosphere for 48 hr. The *C. jejuni* reference strain NCTC 11168 and *C*. *coli* clade 3 isolate 76339 (Skarp‐de Haan et al., 2014) were included for comparison and the experiment was performed in duplicates two or three times for each isolate.

#### 2.3.3 Biofilm formation

Evaluation of the ability of the isolates to form biofilm was performed as previously described by Revez et al. (2011) with minor modifications. Bacteria were harvested from blood agar and resuspended in PBS to a concentration of 4 x 10^6^ cfu/ml. Of the bacterial suspension, 10 μl was added to 1 ml Brucella broth in glass tubes and incubated microaerobically at 37°C. After 48 hr the broth was removed and the tubes were stained with 1% crystal violet solution. The isolates were positive for biofilm formation if a stained band was seen at the air‐liquid interface. The *C*. *coli* clade 1 reference strain LMG 6440 was included for comparison, the *C. jejuni* isolate 76577, previously identified as positive for biofilm formation (Revez et al., 2011), was used as a positive control and broth without addition of bacteria was used as a negative control.

#### 2.3.4 Motility

Motility assays were performed according to Szymanski et al. (1995), with a few modifications. A bacterial suspension in PBS with the concentration of approximately 10^8^ cfu/ml was prepared as described previously. Of the bacterial suspension, 5 μl was stabbed into a Brucella soft agar plate (0.4%) and swarming zones were measured after incubation at 42°C in microaerobic atmosphere for 48 hr. The *C*. *coli* clade 1 reference strain LMG 6440 was included for comparison.

#### 2.3.5 Colony morphology

The isolates were cultured on blood and CCDA plates for 48 hr at 42°C in microaerobic atmosphere. The morphology of the colonies on agar plate was analyzed visually.

#### 2.3.6 Use of tricarballylate

An inoculum with a starting concentration of 10^6^ cfu/ml was prepared in nonsupplemented and with 20 mmol/L tricarballylate (Sigma‐Aldrich, St Louis, MO, US) supplemented RPMI 1640 media. Each media was adjusted with sodium hydroxide to pH 7. RPMI 1640 without supplements served as a negative control and inoculated Brucella broth served as a positive growth control. Cultures were incubated microaerobically at 42°C. Growth was monitored over 24 hr by measuring the OD_405_ with Novaspec II (formerly Pharmacia LKB Biotechnology, Uppsala, Sweden) at the start and after 24 hr.

### 2.4 Statistical analyses

The unpaired *t* test was used to assess differences between clade 2 (*n* = 5) and clade 3 (*n* = 3) groups in motility, survival in water, and use of tricarballylate, respectively. A *p*‐value <.05 was considered significant.

## 3 RESULTS

### 3.1 *C*. *coli* water isolates belonged to clades 2 and 3

**Figure 1:**
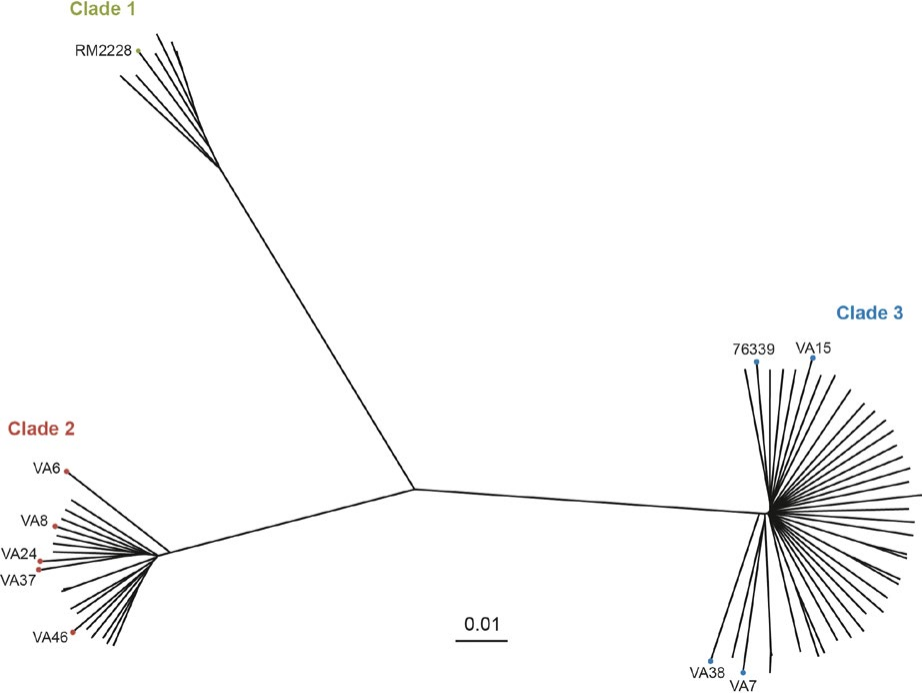
Neighbour‐joining tree with clade division based on phylogenetic analysis of the whole genomes of the following isolates: eight Swedish *Campylobacter coli* water isolates (VA), 53 additional publicly available clade 2 and 3 water isolates, the clinical *C*. *coli* clade 3 isolate 76339 (Skarp‐de Haan et al., 2014), the *C*. *coli* clade 1 reference strain RM2228 and clade 1 isolates from a previously published collection (Sheppard et al., 2013)

Eight *C*. *coli* isolates, collected from raw (incoming) surface water at water plants in Sweden, were whole‐genome sequenced (Table 1; GenBank Bioproject number PRJNA353352). Phylogenetic analyses of the whole‐genome sequences of the *C*. *coli* water isolates were performed together with 53 additional *C*. *coli* clade 2 (*n* = 16) and clade 3 (*n* = 37) sequences from the NCBI database, originating from isolates cultured from environmental waters, and the sequences of the clinical clade 3 isolate 76339 (Skarp‐de Haan et al., 2014), the *C*. *coli* clade 1 reference strain RM2228 and *C*. *coli* clade 1 isolates from a previously published collection (Sheppard et al., 2013). The results showed that the Swedish water isolates were divided into distinct clades; where five of the isolates belonged to clade 2 and three to clade 3, respectively (Figure 1 and Table 1). None of these eight *C*. *coli* isolates could be assigned to a known sequence type or clonal complex at the time of the analysis.

### 3.2 Clade 2 isolates had significantly larger genomes

On average, more genetic content was found in clade 2 than in clade 3 of the Swedish *C*. *coli* water isolates, including small plasmids in four out of five clade 2 isolates (Table 1). This was also true for all clade 2 (mean size = 1.79 Mbp) and clade 3 (mean size = 1.59 Mbp) isolates when the additional 53 earlier published whole‐genome sequences were included (*p* < .0001, Table S1 and data not shown).

### 3.3 Genes not shared by clade 2 and clade 3

Comparative genomics analyses of Swedish water isolates detected a total of 1752 orthologous groups, of which 1430 were shared between the two clades. A total of 73 orthologous groups were only found in the *C*. *coli* clade 2 water isolates, whereas the corresponding number was 29 for *C*. *coli* clade 3 water isolates (Table S2). Of the 73 ortholog groups only found in the clade 2 water isolates, 40 were assigned a putative function, whereas 33 were hypothetical. For the ortholog groups only found in the *C*. *coli* clade 3 water isolates, 21 were assigned a putative function and 8 were hypothetical. A majority of these ortholog groups, with putative functions and only detected in one of the two clades, were also verified in the 53 additional *C*. *coli* clade 2 and 3 sequences from the NCBI database (Table S2). Comparison of these clade‐specific orthologous groups revealed a wide variety of carbon metabolism‐related coding sequences in clade 2, whereas in clade 3 genes involved in oxidative stress and putative virulence factors were found (Table S2). In addition, the clade 2 water isolate VA46 contained an intact Type VI secretion system (T6SS) locus.

### 3.4 Tricarballylate utilization locus identified in clade 2 isolates

All Swedish clade 2 water isolates possessed a tricarballylate utilization locus consisting of three genes (*tcuCAB*) and a possible transcriptional regulator (R). A BLASTn analysis of the three *tcuCAB* genes against all bacterial sequences within the NCBI database only revealed one hit in *Campylobacter cuniculorum* LMG 24588*,* with a 78% sequence similarity. In addition, BLAST searches in Bionumerics performed on the 53 additional *C*. *coli* clade 2 and 3 sequences from the NCBI database and *C*. *coli* clade 1 sequences from a previously published collection (Sheppard et al., 2013) identified the full *tcuRCAB* locus (including the regulator) only in the *C*. *coli* clade 2 sequences and in two of the clade 1 sequences (BIGS0005 and BIGS0021). However, the sequence of the regulator in the *C. cuniculorum* strain LMG 24588 contained a premature stop codon. Alignment and phylogenetic analysis of *tcuRCAB* sequences revealed high sequence similarity between all clade 2 isolates (Figure S1a). The clade 1 isolate (BIGS0005) was placed close to the clade 2 isolates, but on a separate branch (Figure S1a). The *C. cuniculorum* LMG 24588 was placed much further away on the tree indicating a big sequence difference to the *C*. *coli tcuRCAB* genes. A BLASTp analysis of the *tcuCAB* locus against the NCBI database was also performed and yielded no hits for the full locus, with or without the possible transcriptional regulator included.

### 3.5 Putative citrate transporter, motility, and *pseA* genes

A putative citrate transporter was identified among all Swedish clade 3 water isolates, whereas only present in one clade 2 isolate (VA8) as a highly fragmented open reading frame (ORF) (data not shown). A BLASTn of the putative citrate transporter against all *Campylobacter* sequences in the NCBI database yielded hits in the clinical *C*. *coli* clade 3 isolate 76339. A further search in the additional clade 2 and 3 sequences included in the genome analyses identified the gene sequence of the putative citrate transporter in all *C*. *coli* clade 3 sequences and also in four of the *C*. *coli* clade 2 sequences.

All major motility genes, such as *flaA*,* flaB, motA, motB, flh, flg,* and *fli*, were present among all the water *C*. *coli* isolates. Also, *flhA* was present as an intact ORF in our isolates. Alignment and phylogenetic analysis of the sequences of the *motA* and *motB* genes identified in the Swedish *C*. *coli* water isolates, the clinical *C*. *coli* clade 3 isolate 76339, *C*. *coli* clade 1 reference strain RM2228, *C*. *coli* clade 1 from a previously published collection (Sheppard et al., 2013) and the included *C*. *coli* clade 2 and 3 sequences from environmental water isolates revealed a clade‐specific division of the isolates with a high sequence similarity within the clades (Figure S1b).

*PseA*, involved in flagella biosynthesis, was found in all Swedish clade 3 isolates, in most of the additional clade 3 sequences included in the genomic analysis and in selected clade 1 sequences (Sheppard et al., 2013) (Table S1). Alignment and phylogenetic analysis of the *pseA* gene sequences showed a clear clade‐specific division between clades 1 and 3 (Figure S1c).

### 3.6 Differences in water survival, colony morphology, and motility between clade 2 and 3 water isolates

**Figure 2:**
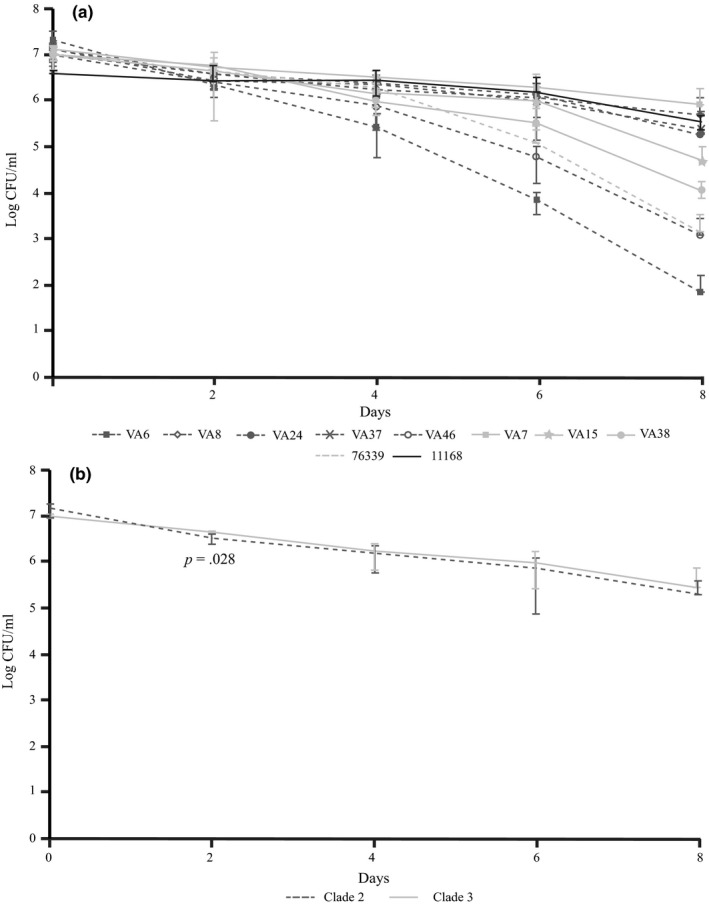
(a) Survival in autoclaved tap water of *C*. *coli* water isolates (*n* = 8), the clinical *C*. *coli* clade 3 isolate 76339 and *Campylobacter jejuni* NCTC 11168 reference strain as monitored over 8 days. (Darker dashed lines, clade 2 isolates; lighter lines, clade 3 isolates). Mean survival with error bars indicating *SD*s are shown. (b) Water survival in autoclaved tap water of the *C*. *coli* water isolates grouped according to the clades (clade 2, *n* = 5; clade 3, *n* = 3). Mean survival of isolates in each group with error bars indicating *SD*s are shown

The water survival of the eight *C*. *coli* water isolates, the clinical *C*. *coli* clade 3 isolate 76339 and *C. jejuni* NCTC 11168 was monitored over 8 days (Figure 2a). The survival was calculated from the number of culturable bacteria at days 0, 2, 4, 6, and 8. The survival varied slightly between the isolates, and a significant difference could be seen between *C*. *coli* clade 2 and 3 water isolates on day 2 when clade 3 isolates showed a higher survival (*p* = .028) (Figure 2b). The clade 2 isolate VA6 showed the lowest survival of all the isolates tested at all time points (Figure 2a).

Apart from the positive control *C. jejuni* 76577, none of the *C*. *coli* formed biofilm in three independent experiments (data not shown).

**Figure 3:**
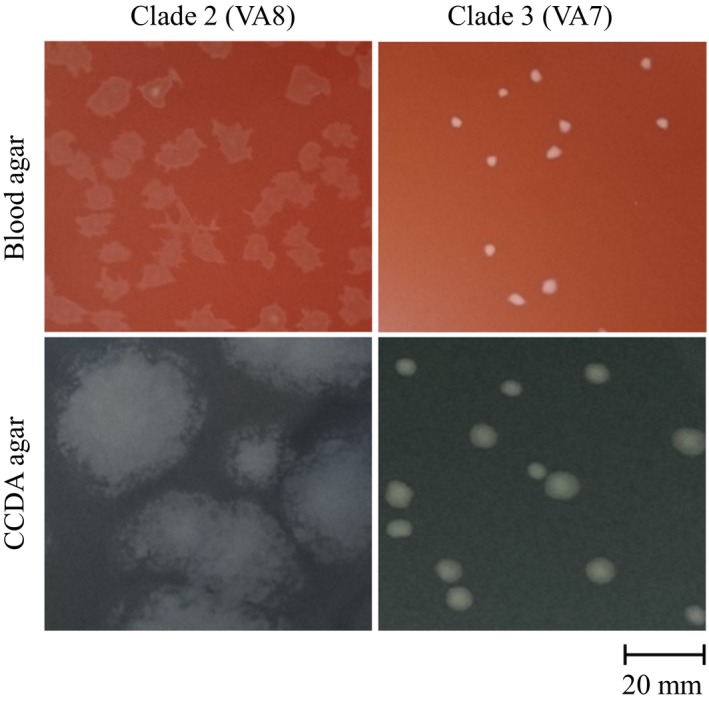
Colony morphology of *C*. *coli* clade 2 (VA 8) and clade 3 (VA 7) isolates on blood and CCDA agar

Colony morphology was assessed on blood and CCDA agar. All clade 3 isolates and one clade 2 isolate, VA6, formed round and even colonies (Figure 3). In contrast, the rest of the clade 2 isolates formed large and blurry colonies difficult to distinguish from each other (Figure 3).

**Figure 4:**
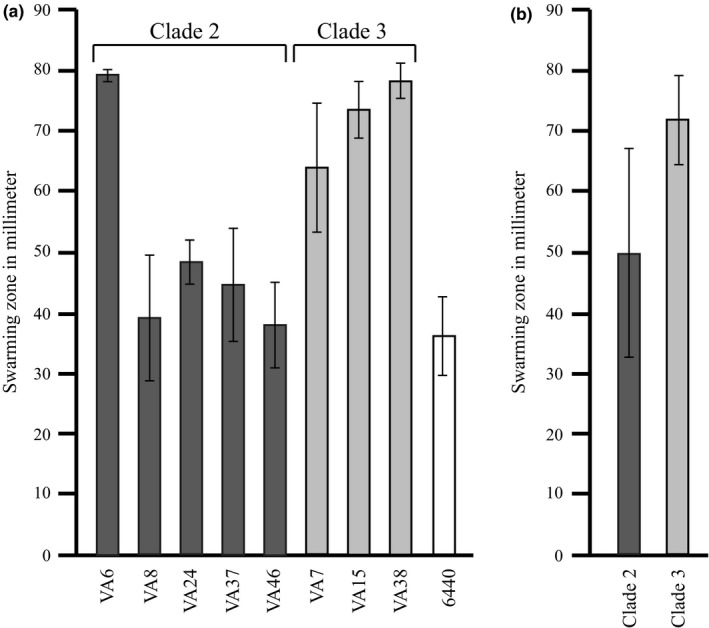
Motility shown as swarming diameters in soft agar plates. (a) The motility of the *C*. *coli* water isolates (*n* = 8) and the *C*. *coli* clade 1 reference strain LMG 6440. Mean values of 3 experiments with error bars indicating *SD*s are shown. (b) The motility of the *C*. *coli* water isolates grouped together according to the clades (clade 2, *n* = 5; clade 3, *n* = 3). Mean values of isolates in each group with error bars indicating *SD*s are shown

When the motility of the *C*. *coli* water isolates was tested (Figure 4a), the clade 3 *C*. *coli* water isolates showed larger swarming zones than the clade 2 water isolates although this difference was not significant (*p* = .085) (Figure 4b). However, one clade 2 isolate, VA6, showed a motility similar to clade 3 isolates (Figure 4a).

### 3.7 Clade 2 isolates able to utilize tricarballylate

**Figure 5:**
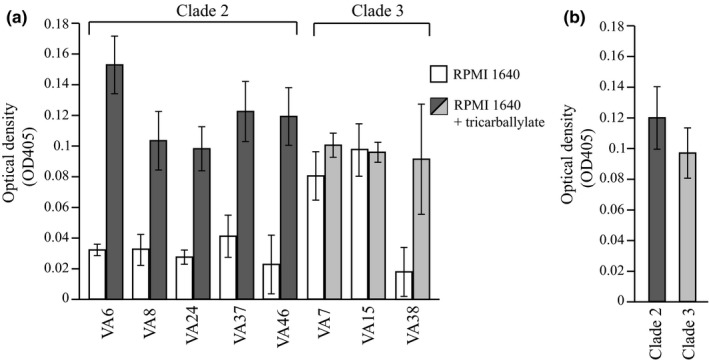
(a) Growth of the *C*. *coli* water isolates (*n* = 8) after 24 hr in nonsupplemented RPMI 1640 medium (white bars) and in RPMI 1640 medium supplemented with 20 mmol/L tricarballylate (gray bars). Mean values of 3 experiments with error bars indicating *SD*s are shown. (b) Growth in RPMI 1640 medium supplemented with 20 mmol/L tricarballylate of the *C*. *coli* water isolates grouped together according to the clades (clade 2, *n* = 5; clade 3, *n* = 3). Mean values of isolates in each group with error bars indicating *SD*s are shown

As a putative tricarballylate utilization locus was found in the clade 2 isolates, all *C*. *coli* water isolates were tested for the ability to use tricarballylate as an energy source. All clade 2 isolates grew clearly better in RPMI 1640 media supplemented with tricarballylate than in nonsupplemented media (Figure 5a). In contrast, the clade 3 isolates did not grow better in the supplemented media, with exception for the isolate VA38, which grew as the clade 2 isolates (Figure 5a). However, there was no significant difference between the clade 2 and 3 isolates in the supplemented media (Figure 5b), as two of the latter showed good growth even in the nonsupplemented media (Figure 5a).

## 4 DISCUSSION

In this study, whole‐genome sequencing was combined with various phenotypic analyses in order to characterize *C*. *coli* isolates from raw surface water at water plants at different locations in Sweden. Our results showed that the eight *C*. *coli* isolates belonged to clades 2 and 3 and these two clades have been suggested to be common among environmental *C*. *coli* (Sheppard et al., 2013). Our aim was to detect unique traits among these isolates to further understand and highlight the diversity of *C*. *coli* in general and possibly define features that could explain the ability of these particular isolates to survive in water. As our collection of *C*. *coli* water isolates was limited, 53 additional earlier published sequences of clade 2 and 3 water isolates were included for comparisons. However, the results obtained in phenotypic analyses were still based on a low number of isolates.

Clade 2 isolates had significantly larger genomes than those of clade 3, and comparative genomic analysis revealed that *C*. *coli* clade 2 water isolates contained genes for various metabolic capabilities in contrast to *C*. *coli* clade 3 isolates. One of these features, a putative tricarballylate gene locus, was further subjected to phenotypic characterization. As expected from the genomic annotation, the clade 2 isolates grew clearly better in RPMI 1640 media supplemented with tricarballylate than in media without tricarballylate, but surprisingly also clade 3 isolate VA38 could use this carbon source. This indicates that affinity for certain carbon sources exists and that this may vary between different types of isolates. Tricarballylate is the causative agent of grass tetany (magnesium deficiency) in ruminants and is formed by rumen microorganisms as a fermentation product of transaconitate (Lewis, Horswill, Schwem, & Escalante‐Semerena, 2004; Russell, 1985). Earlier, *Salmonella enterica* serovar Typhimurium, a serotype associated with a broad host range, was shown to encode a functional tricarballylate metabolism (Lewis et al., 2004). It is tempting to speculate that the use of tricarballylate as an energy source may be advantageous in the colonization of ruminants.

Most water survival studies for *Campylobacter* have been performed using *C. jejuni* isolates and survival times have ranged from 2 weeks to 4 months (Cools et al., 2003; Rollins & Colwell, 1986; Trigui et al., 2015). In several studies, autoclaved tap water has been used to obtain sample reproducibility and to avoid influence of native water microbiota (Bronowski et al., 2017; Buswell et al., 1998). Here, the short‐term survival of all the Swedish *C*. *coli* water isolates was analyzed in autoclaved tap water. Also, the experiments were performed at 4°C as a low temperature has been suggested to promote survival of *Campylobacter* in different types of water (Thomas et al., 1999). Our local tap water, used in the experiment, is chlorinated to prevent bacterial growth, however, autoclaving decomposes sodium hypochlorite, which therefore should not affect the bacterial survival at the time of the analysis. In accordance with previous reports (Buswell et al., 1998; Thomas et al., 1999), most of our *C*. *coli* isolates had a lower survival than the *C. jejuni* reference strain NCTC 11168. However, a more extended survival comparison would elucidate whether this is also true for *C. jejuni* water isolates.

None of our *C*. *coli* clade 2 and 3 isolates were able to produce biofilm under the conditions tested, which could indicate that no intrinsic biofilm formation capability exists within these clades. Other studies have suggested that different genetic factors, such as motility and glycosylation of flagellar structures, may influence biofilm formation in *C. jejuni* (Oh & Jeon, 2014; Pascoe et al., 2015). The *C*. *coli* clade 2 and 3 water isolates and the clade 1 reference strain LMG 6440 were motile, but the clade 3 isolates and the clade 2 isolate VA6 were hypermotile as compared to the other clade 2 water isolates and LMG 6440. As all motility genes including flhA (Park, Purdy, & Leach, 2000) were present in all *C*. *coli* water isolates this could not explain the difference between the clades. Phylogenetic analysis of the *motA* and *motB* gene sequences revealed differences between the clades (Figure S1b), however, the impact of these differences is unknown. *PseA* was found in most of the clade 3 sequences but in none of the clade 2 sequences analyzed (Table S2) and is involved in the formation of the acetamidino form of pseudaminic acid (PseAm) in the N‐linked glycosylation pathway of the flagella. Although loss of PseAm has not resulted in loss of motility in *C. jejuni* (Guerry et al., 2006), we speculate that the absence of *pseA* among the clade 2 water isolates could partly explain their lower motility.

Differences in motility may also be reflected in colony appearance on agar plates (Reuter & van Vliet, 2013). Colony appearance may also be due to phase variation in different structures involved in cellular or flagellar biosynthesis (Park et al., 2000). This would at least explain the opposite phenotype of the clade 2 isolate VA6, which in both motility and colony appearance resembled clade 3 isolates. However, as the difference between the clades was consistent, it is unlikely that it can only be explained by phase variation, as more variation between the experiments would have been observed. One feature present in clade 3, a putative MCP‐type signal transduction protein, though piqued our interest, as a BLASTp search with the tCDS found 87% similarity to the Cj1110c gene (*tlp8*) of *C. jejuni* NCTC 11168. Also, in the additional *C*. *coli* clade 1, 2, and 3 whole‐genome sequences included, the *tlp8* sequence was only identified among the clade 3 sequences. Cj1110c mutants have shown attenuation both in their colony appearance and in energy taxis assays. Interestingly and in contrast to Reuter & van Vliet (2013) who showed increased swarming for *tlp8* mutant *C. jejuni* strains, our *C*. *coli* clade 2 isolates (lacking *tlp8*) demonstrated a smaller swarming zone in the motility assay. Moreover, Tlp8 mediates movement away from an environment with a high oxygen tension to an environment with more microaerobic conditions. Theoretically, clade 3 *C*. *coli* isolates possessing features such as Tlp8, cytochromes, and the DMSO reductase system, which could aid in reducing the oxygen tension, would be better to handle oxidative stress. In contrast, clade 2 isolates seem to exhibit a more extensive TCA cycle metabolism, for which oxygen is required and thus may be less affected by fluctuating oxygen tensions.

## 5 CONCLUSIONS

The eight Swedish *C*. *coli* water isolates were assigned to the clades 2 and 3, which have earlier been associated with environmental origins. The combined results from comparative genomics and phenotypical analyses as well as in silico analyses using earlier published sequences suggest differences in metabolic capabilities and ways to handle oxidative stress between clade 2 and clade 3 isolates.

## **CONFLICT OF INTERESTS**

The authors declare that they have no competing interests.

## **ETHICS STATEMENT**

This research did not involve any human or animal subjects, materials, or data and therefore did not require any ethics oversight or approval in these respects.
