# Supplementary figures and images for: Characterization of Swedish Campylobacter coli clade 2 and clade 3 water isolates
Source: Microbiologyopen. 2018 Feb 9;7(4):e00583. doi: 10.1002/mbo3.583 (PMC6079167; doi:10.1002/mbo3.583)

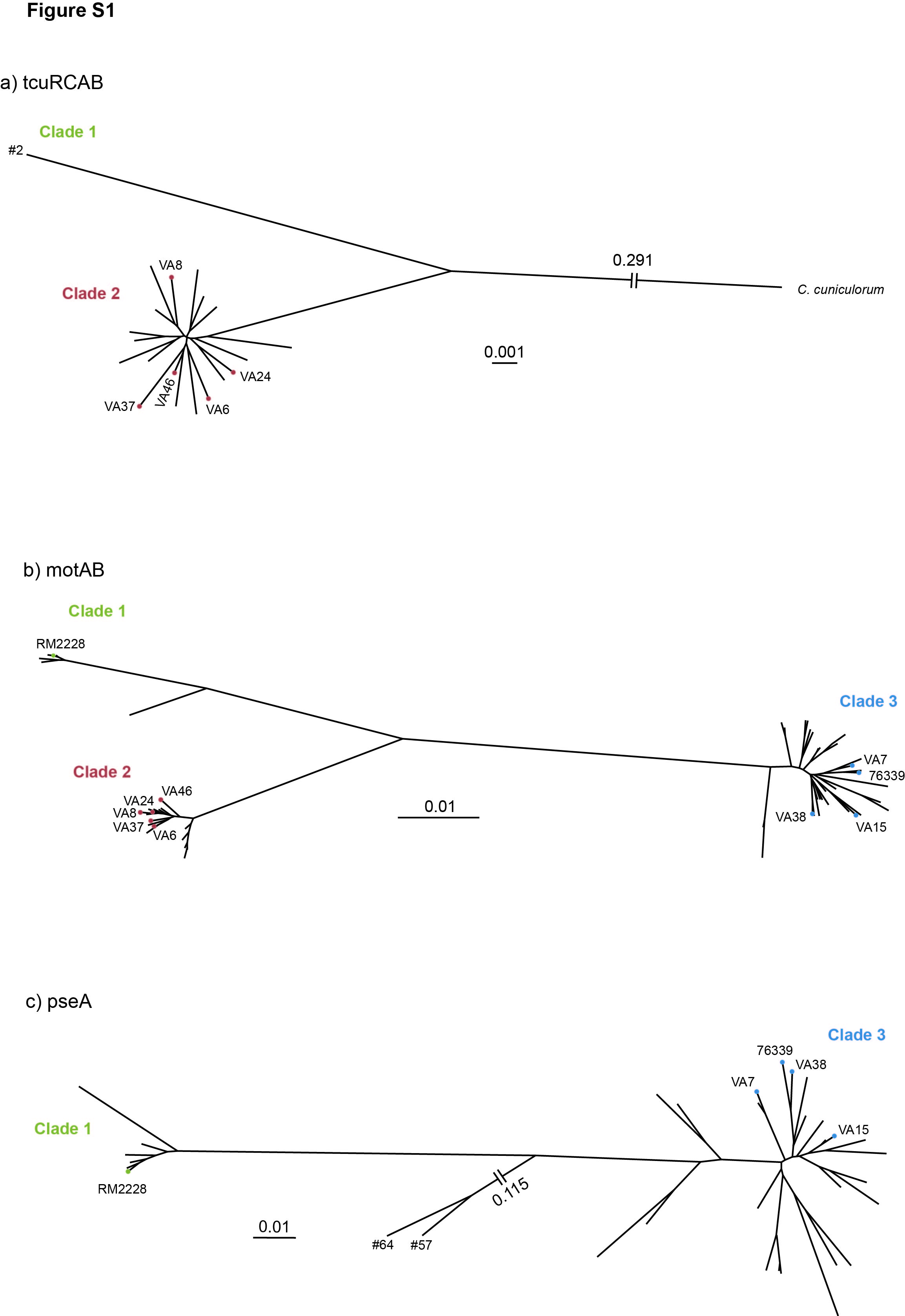

Supplement: Supplementary file 1 [file MBO3-7-e00583-s001.jpg]
