# Supplementary material for: Characterization of Swedish Campylobacter coli clade 2 and clade 3 water isolates
Source: Microbiologyopen. 2018 Feb 9;7(4):e00583. doi: 10.1002/mbo3.583 (PMC6079167; doi:10.1002/mbo3.583)
Supplement: Supplementary file 2 [file MBO3-7-e00583-s002.docx]

Table S1. NCBI sequences included in genomic analyses.

| **Nr** | **Clade** | **Isolate** | **Source** | **NCBI**  **accession number** | **Genome size**  **(Mbp)** |
| --- | --- | --- | --- | --- | --- |
| 1 | 1 | BIGS2 | Clinical | ANGM01 | 1.68 |
| 2 | 1 | BIGS5 | Pig faeces | ANGP01 | 1.59 |
| 3 | 1 | BIGS15 | Pig faeces | ANGZ01 | 1.59 |
| 4 | 1 | BIGS17 | Chicken meat | ANHB01 | 1.63 |
| 5 | 1 | BIGS19 | Clinical | ANHD01 | 1.68 |
| 6 | 1 | BIGS21 | Chicken meat | ANHF01 | 1.57 |
| 7 | 1 | RM2228 | Reference strain | AAFL01 | 1.86 |
| 8 | 2 | VA6 | Environmental waters | MPIQ01 | 1.76 |
| 9 | 2 | VA8 | Environmental waters | MPIS01 | 1.83 |
| 10 | 2 | VA24 | Environmental waters | MPIU01 | 1.83 |
| 11 | 2 | VA37 | Environmental waters | MPIV01 | 1.86 |
| 12 | 2 | VA46 | Environmental waters | MPIX01 | 1.95 |
| 13 | 2 | A12B2 | Environmental waters | FBGG01 | 1.78 |
| 14 | 2 | B15B3 | Environmental waters | FBFU01 | 1.77 |
| 15 | 2 | F14B4C4 | Environmental waters | FBGM01 | 1.85 |
| 16 | 2 | F15D2 | Environmental waters | FBFW01 | 1.79 |
| 17 | 2 | H055260513 | Environmental waters | FBNY01 | 1.73 |
| 18 | 2 | H055260517 | Environmental waters | FAXG01 | 1.78 |
| 19 | 2 | H091320798 | Environmental waters | FBKV01 | 1.73 |
| 20 | 2 | H091940890 | Environmental waters | FBOS01 | 1.73 |
| 21 | 2 | H092560131 | Environmental waters | FBKJ01 | 1.75 |
| 22 | 2 | H093580632 | Environmental waters | FBPP01 | 1.8 |
| 23 | 2 | H094560718 | Environmental waters | FBKY01 | 1.74 |
| 24 | 2 | H105160209 | Environmental waters | FBQJ01 | 1.73 |
| 25 | 2 | K11 | Environmental waters | FBGA01 | 1.73 |
| 26 | 2 | M2D2 | Environmental waters | FBGB01 | 1.81 |
| 27 | 2 | M4A4 | Environmental waters | FBGH01 | 1.8 |
| 28 | 2 | M5D4 | Environmental waters | FBQV01 | 1.83 |
| 29 | 3 | VA7 | Environmental waters | MPIR01 | 1.66 |
| 30 | 3 | VA15 | Environmental waters | MPIT01 | 1.68 |
| 31 | 3 | VA38 | Environmental waters | MPIW01 | 1.77 |
| 32 | 3 | 76339 | Clinical | HG326877 | 1.58 |
| 33 | 3 | BIGS3 | Environmental waters | ANGN01 | 1.62 |
| 34 | 3 | C9A1 | Environmental waters | FBQZ01 | 1.6 |
| 35 | 3 | D13A4 | Environmental waters | FBFX01 | 1.59 |
| 36 | 3 | H053120426 | Environmental waters | FBMY01 | 1.61 |
| 37 | 3 | H060740717 | Environmental waters | FBIP01 | 1.57 |
| 38 | 3 | H060960756 | Environmental waters | FBGE01 | 1.56 |
| 39 | 3 | H060960757 | Environmental waters | FBHG01 | 1.56 |
| 40 | 3 | H061680633 | Environmental waters | FBOE01 | 1.58 |
| 41 | 3 | H061980521b | Environmental waters | FBNK01 | 1.6 |
| 42 | 3 | H065100506 | Environmental waters | FBKP01 | 1.58 |
| 43 | 3 | H065160534 | Environmental waters | FBJV01 | 1.58 |
| 44 | 3 | H071960681 | Environmental waters | FBNO01 | 1.57 |
| 45 | 3 | H072620566 | Environmental waters | FBKO01 | 1.59 |
| 46 | 3 | H073220518 | Environmental waters | FBNQ01 | 1.59 |
| 47 | 3 | H073580401 | Environmental waters | FBAL01 | 1.63 |
| 48 | 3 | H074080509 | Environmental waters | FBOA01 | 1.6 |
| 49 | 3 | H074080511 | Environmental waters | FBNP01 | 1.58 |
| 50 | 3 | H075140555 | Environmental waters | FBKA01 | 1.59 |
| 51 | 3 | H075200522 | Environmental waters | FBMZ01 | 1.57 |
| 52 | 3 | H081380695a | Environmental waters | FBNU01 | 1.59 |
| 53 | 3 | H082280513 | Environmental waters | FAZO01 | 1.61 |
| 54 | 3 | H082280515 | Environmental waters | FAZW01 | 1.57 |
| 55 | 3 | H082560569 | Environmental waters | FAYV01 | 1.63 |
| 56 | 3 | H083420694 | Environmental waters | FBNG01 | 1.58 |
| 57 | 3 | H083420701 | Environmental waters | FBNT01 | 1.57 |
| 58 | 3 | H085160742 | Environmental waters | FBJZ01 | 1.58 |
| 59 | 3 | H085160749 | Environmental waters | FBLD01 | 1.62 |
| 60 | 3 | H090980249 | Environmental waters | FBKL01 | 1.57 |
| 61 | 3 | H092260569b | Environmental waters | FBNM01 | 1.56 |
| 62 | 3 | H093580324 | Environmental waters | FBKN01 | 1.58 |
| 63 | 3 | H094560713 | Environmental waters | FBKD01 | 1.57 |
| 64 | 3 | H094560720 | Environmental waters | FBKQ01 | 1.56 |
| 65 | 3 | H094860392 | Environmental waters | FBAT01 | 1.55 |
| 66 | 3 | H102740169 | Environmental waters | FBPR01 | 1.62 |
| 67 | 3 | H110340458 | Environmental waters | FBQC01 | 1.6 |
| 68 | 3 | H132840800 | Environmental waters | FBPJ01 | 1.55 |
| 69 | 3 | K3D1 | Environmental waters | FBFT01 | 1.56 |

Table S2. RAST annotations of orthologues only present in clade 2 or clade 3 among Swedish *Campylobacter coli* water isolates, respectively. The presence/absence of corresponding orthologues is shown for other published sequences of clade 2 and clade 3 isolates from environmental waters.

| **RAST annotation**  **(number of features in parenthesis)** | **Found in other published sequences^a,b^** |
| --- | --- |
| **Only present in clade 2 among our *C. coli* water isolates** |  |
| Hypothetical protein (23) | Not verified |
| Hypothetical protein^c^ (10) | Not verified |
| Aliphatic amidase AmiE (1) | All |
| Alpha-2-macroglobulin (1) | All |
| L-carnitine dehydratase/bile acid-inducible protein F (1) | All |
| CfrA (1) | All but also found in clade 3 sequences 32-35, 40-41, 45-46, 48, 50-53, 56-57, 60, 62, 64-65 and 67. |
| Citrate lyase β chain (1) | All |
| Cj0413 homolog (1) | All |
| COG3777 (1) | All |
| Conserved hypothetical membrane protein^c^ (1) | All except 24 and 26. |
| Cyclopropane-fatty-acyl-phospholipid synthase (1) | All |
| Dicarboxylic acid transporter PcaT (1) | All but also found in clade 3 sequence 65. |
| F-box DNA helicase I (1) | All except 15, 25, 27 and 28. Also found in clade 3 sequence 55. |
| Hemerythrin-like iron-binding protein (2) | All |
| Inner membrane protein CreD-like protein (1) | All |
| Lipopolysaccharide core biosynthesis protein LpsA (1) | All but also found in clade 3 sequences 34, 57, 59, 64, 66 and 68. |
| Methionine ABC transporter (1) | All |
| Methionyl-tRNA formyltransferase (1) | All but also found in clade 3 sequences 33, 35, 42-43, 45, 47, 49, 56-57 and 69. |
| Outer membrane protein romA (1) | All |
| 2-oxobutyrate oxidase (1) | All |
| 2-oxoglutarate/malate translocator (1) | All |
| Penicillin-insensitive transglycosylase & transpeptidase PBP-1C (1) | All |
| Possible acetyltransferase (1) | All |
| Predicted D-lactate dehydrogenase (1) | All |
| Putative cytochrome C-type haem-binding periplasmic protein (1) | All |
| Putative H-T-H containing protein^c^ (1) | All except 14. |
| Putative integral membrane protein (4) | All |
| Putative membrane protein (2) | All |
| Putative metal chaperone, involved in Zn homeostasis, GTPase of COG0523 family (1) | All |
| Putative polysaccharide deacetylase (1) | All |
| Ribosomal RNA adenine demethylase (1) | All |
| TcuA (1) | All |
| TcuB (1) | All |
| TcuC (1) | All |
| Tgh088 (1) | All |
| Tgh102^c^ (1) | All |
| Transcriptional regulator, GntR family (1) | All |
|  |  |
| **Only present in clade 3 among our *C. coli* water isolates** |  |
| Hypothetical protein (8) | Not verified |
| ABC transporter permease protein (1) | All |
| Anaerobic dimethyl sulfoxide reductase chain A^d^ (1) | All |
| Anaerobic dimethyl sulfoxide reductase chain B^d^ (1) | All |
| Anaerobic dimethyl sulfoxide reductase chain C^d^ (1) | All |
| CMP-N-acetylneuraminate-beta-galactosamide-alpha-2,3-sialyltransferase^d, e^ (1) | None except 45, 49, 50 and 51. |
| Cytochrome C family protein^d^ (1) | All except 57. |
| Ferric iron ABC transporter, ATP-binding (1) | All |
| Ferric iron ABC transporter, iron-binding (1) | All |
| Hemerythrin-like iron-binding protein (1) | All |
| IgA1 protease/autotransporter domain, T5aSS type secretion^d^ (1) | All except 62 |
| Imidazole glycerol phosphate synthase amidotransferase subunit (1) | All except 33, 35, 42-43, 45, 47, 49, 56 and 69. |
| Imidazole glycerol phosphate synthase cyclase subunit (1) | All except 32-33, 35, 42-43, 45, 47, 49, 56 and 69. |
| Phosphoserine phosphatase (1) | All |
| PseA (1) | All except 33, 35, 42, 43, 45, 47, 49, 56 and 69. |
| Putative cytochrome C-type haem-binding periplasmic protein (1) | All |
| Putative MCP-type signal transduction protein (87% similarity with Cj1110c) (1) | All |
| Putative periplasmic protein (1) | All |
| Putative phosphatase (1) | All |
| Putative secreted serine protease^d^ (1) | All |
| Thiamin ABC transporter (1) | All |
| UPF0141 membrane protein YijP possibly required for phosphoethanolamine modification of lipopolysaccharide^e^ (1) | None except 45 and 51. |

^a^Numbers refer to the sequence numbers from Table S1.

^b^All indicates that the feature was found in all clade 2 or clade 3 sequences, respectively.

^c^Absent in VA6. Possibly plasmid derived genetic content.

^d^Previously described as unique clade 3 orthologues (Skarp-de Haan et al., 2014).

^e^Absent in VA38.
